# An English vocabulary learning support system for the learner’s sustainable motivation

**DOI:** 10.1186/s40064-015-0792-2

**Published:** 2015-02-27

**Authors:** Tatsuhito Hasegawa, Makoto Koshino, Hiromi Ban

**Affiliations:** Graduate School of Natural Science and Technology, Kanazawa University, Kakumamachi, Kanazawa, Ishikawa Japan; Department of Electronics and Information Engineering, Ishikawa National College of Technology, Kitachujo, Tsubatamachi, Ishikawa Japan; Faculty of Engineering, Nagaoka University of Technology, Kamitomioka, Nagaoka, Niigata Japan

**Keywords:** Vocabulary learning, Sustainable motivation, Gamification

## Abstract

In English vocabulary learning, continuation is an important factor; however, many learners are not good at continuing learning because they tend to prefer amusement or rest. Our proposed system is targeting learners who are eager to learn but are not able to continue learning for various reasons. We especially focused on English vocabulary learning, and described an approach for learners who have difficulty with continuing learning. Our developed application aggressively supports the learners’ sustainable motivation by gamification techniques and an efficient difficulty setting method.

## Introduction

The term “electronic learning,” that is, “e-learning,” has become popular since the mid-1990s, and its goal is to create a community of inquiry independent of time and location through information and communication technologies Garrison (2011). Currently, companies and researchers are developing various types of e-learning systems. Because of the recent remarkable popularization and improvement in mobile devices all over the world, many people carry their smartphone on a daily basis; therefore, they can easily use ICT-technologies such as a telephone call, e-mail, accessing the internet, and various applications at all times. Accordingly, considering an e-learning environment where mobile devices are used, the term “mobile learning,” or m-learning, is also focused on Cavus and Ibrahim (2009). According to a practical study Evans (2008) which utilized m-learning in the form of podcasting, students believe that the podcasts are more effective than their textbooks as revision tools, and that they are more efficient than their own notes in helping them to learn. We considered that using a smartphone is effective for independent learning such as English vocabulary memorization because a learner can use the smartphone anytime and anywhere when the learner has only a little time. In English vocabulary learning, continuation is an important factor, although, many learners are not good at continuing learning. Among the learners who are not good at continuing to learn, there are two types of learners: (1) learners who have little motivation to learn; (2) learners who cannot resist the temptation as an amusement, even though they have motivation to learn. In this study, focusing on (2) learners, we developed an English vocabulary learning application for the learner’s sustainable motivation by gamification and cloud intelligent techniques utilizing characteristics of smartphones.

## Related works

E-learning has an advantage that a learner can learn alone anytime and anywhere; however, it also has a disadvantage that it is difficult for the learner to maintain his/her motivation. In this section, we clarify a position and a characteristic of our study, through considerations of related works which enhance the learners’ motivation in the e-learning environment.

In order to maintain the learners’ motivation, we focused on gamification techniques. Gamification is not to create a video game, but it means techniques that are used in creating games for amusing the users. Utilizing the techniques in various scenes can enhance the player’s motivation Deterding et al. (2011). A SNS service Foursquare ^a^ succeeded to enhance the users’ motivation to check in their location to the service through gamification techniques that each user can get some points and badges when checking in. In addition, Preira et al. (2014) developed a smartphone application intended to change the user’s behavior through participating in the collaborative game. Therefore, we considered that utilizing gamification techniques in e-learning has a positive influence on the user’s learning motivation.

We considered that difficulty settings of questions are also important to maintain the learner’s motivation. In any subject, when most questions are very difficult, beginner learners will not be able to maintain their motivation owing to their pains caused by that they cannot answer most questions. In contrast, when questions are an appropriate difficulty for each learner, learners can feel a sense of accomplishment and self-growth. We expected that it enhances learners’ motivation. Among works related to the method of appropriate difficulty estimation, Chen et al. (2007) developed a system that recommends appropriate difficulty English articles depending on the learner’s English skill by estimating their skill using fuzzy item response theory (FIRT) on the mobile device.

In this study, focusing on the independent learning of English vocabulary memorization, we developed an m-learning application utilizing gamification techniques for keeping the learner’s motivation. Moreover, we proposed a new method, which estimates appropriate difficulty questions.

## Proposal system design

In this study, we developed an English vocabulary learning support system for the learner’s sustainable motivation ^b^. Although this application does not depend on content type, in this study, we focused on the basic level of TOEIC vocabulary learning. Further, we focused on motivated learners who cannot continue learning, excluding learners who have little motivation to learn. Researchers (Bachen and Raphael 2007; Simoes et al. 2011; Tan et al. 2013) have proposed some design model for applying gamification techniques in educational situations. We designed our system functions referring to the digital game-based learning framework proposed by Tan et al. (2007) because it can be utilized to fulfill our system concept that encourages learners’ motivation or engagement to learn in the m-learning environment. Surveying four game-based learning models, Tan et al. proposed an original framework in which an educational game should be composed of three elements including multimodal, task, and feedback for game design. Multimodal is a variety of interaction or modalities that connects a learner and a game, namely, various sounds, animations, effects, and such interactional activities for enhancing the learner’s enjoyments. Task is a question or problem in the game to help learners to absorb the learning content (i.e., English word questions in this case), and it should be designed with different levels. Task is similar to challenge in the gameflow Bachen and Raphael (2011). In that paper, the authors described: “If an activity is too easy for the player she grows bored, while if it is too hard she grows frustrated”; therefore, an educational game designed as an adaptive game that continually adjusts difficulty levels to individual players’ skills. Feedback for learners is vital in an educational game, and it is emphasized in various design models. A suitable feedback comment reduces the learner’s misunderstandings. Rewards that help the learners in evaluating their assessment give their confidence in learning. Our proposed functions are as follows:

1. Multimodal
  Basic functions including implementing word utterance, incorporating synonym, antonym, and example sentence.
  Growth character system.
2. Task
  Four levels distributed depending on each word’s difficulty.
  Question selection by estimating the learner’s skills.
3. Feedback
  Time trial challenge and ranking system.
  Connecting SNS.
  Visualization of learners’ efforts and degree of memorization.

We describe the details of these functions in the following sections.

### Basic functions

**Figure 1:**
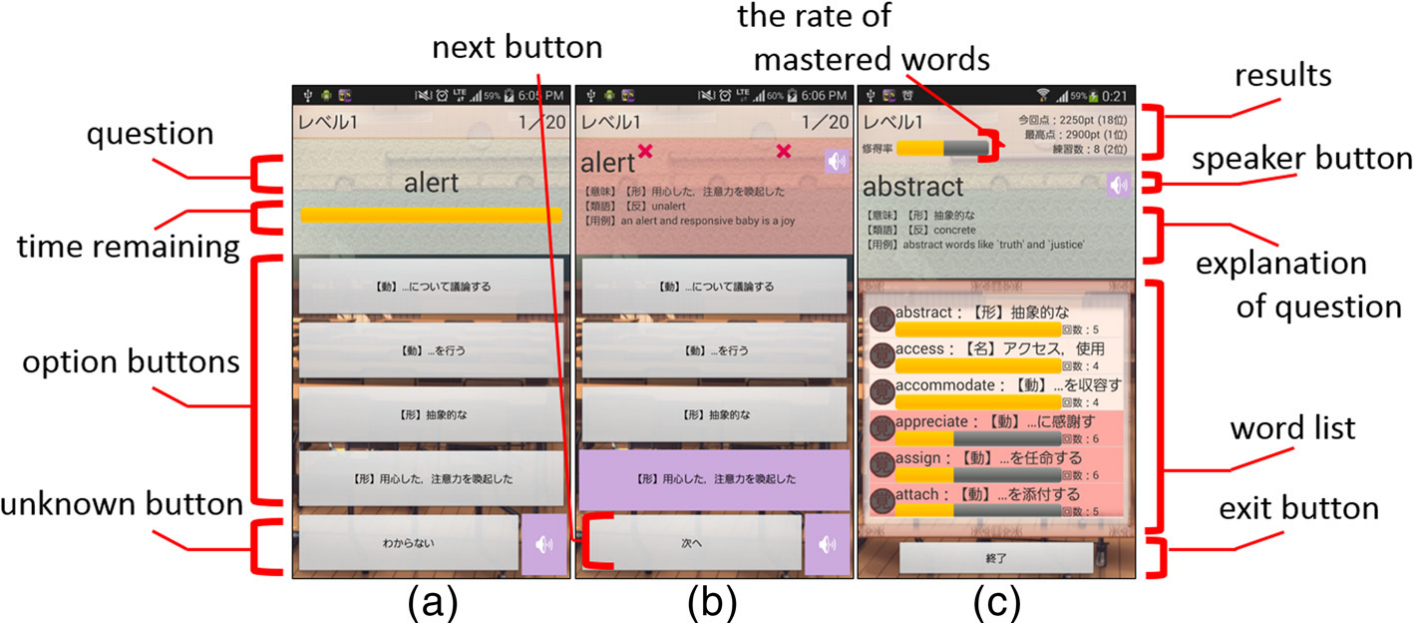
**Activities about learning.** Activities about learning of proposed application: **(a)** Question activity; **(b)** Explanation activity; **(c)** Result list activity.

**Figure 2:**
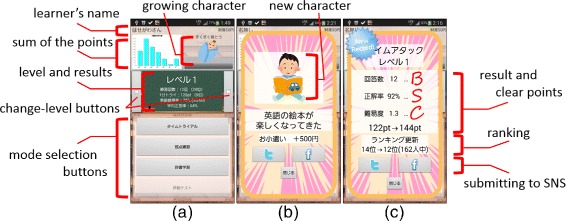
**Activities about gamification techniques.** The pictures related to gamification of our proposed application: **(a)** Operation select activity, **(b)** Event of character growth, **(c)** Result of time trial activity.

Because our application focuses on motivated learners who cannot continue learning, basic function is the learning of simple English vocabulary in a repetition style, as illustrated in Figure 1. Figure 1 (a) shows a question “alert” in the uppermost portion of the screen, four option buttons in the middle of the screen, and the unknown button at the bottom of the screen. The progress bar in the middle of Figure 1 (a) indicates time remaining when playing time trial mode. Figure 1 (b) shows a result and explanation of question including incorporating synonym, antonym, and example sentence in the uppermost portion of the screen. Selecting the option buttons changes this information to the information about the relevant English word. The bottom button indicates the next question. Figure 1 (c) shows all results of this time challenge. A progress bar in the upper left portion of the screen indicates the rate of mastered words in this level. We describe the detail of mastered words below. The information in the upper right portion of the screen shows the result that the learner got in this level: the top line indicates this time challenge, the middle line indicates max points of all challenges, and the bottom line indicates challenged time in this level. When selecting a word in middle words list, the explanation is changed to that for the relevant words. The progress bar for each word is the degree of memorization. We also describe the degree of memorization detail below. When selecting a purple speaker mark, a learner can hear the pronunciation of the question. Figure 2 (a) shows the main screen of our application. The character string in the upper left portion of the screen is the learner’s name which the learner can change any time. The graph in the upper left portion of the screen is the sum of the points the learner gained each day in one week. The image in the upper right portion of the screen is growing character we describe below. The blackboard indicates the question level (in this case level 1) and the result of this level: the top line is the challenged time in this level, the second line is the max points of time trial mode, the third line is the rate of mastered words, and the bottom line is the average rate of the correct answer. The buttons on both sides of the blackboard are change-level buttons. The bottom buttons are for mode selection: first is time trial mode, second is weak point learning mode, third is dictionary mode, and the bottom button is the test mode for challenging to the next level. When the laerner overcome a score in the test mode, the learner can play next level. Because application usability greatly affects learners’ motivation, we enhanced the basic function (e.g., implementing word utterance, incorporating synonym, antonym, and example sentence); further, we incorporated TOEIC basic level 535 words to enhance the contents.

If we create an enjoyable game with a few learning features for keeping learners’ motivation, learners who like video games may continue learning enjoyably. For example, in the RPG, a player can operate a character, can battle enemies, and can collect equipment; further, the player often needs to answer some English vocabulary questions to continue with the story. This idea may be effective for learners who have little motivation to learn; however, the game with a few learning features has disadvantages for our concept as follows:

(1) it increases wasteful time for learning; (2) learners will get tired of learning if the learners get tired of the video game; (3) the pain of learning in itself is not decreased; therefore, in our application, we selected simple English vocabulary learning in a repetition style with some gamification factors.

### Gamifying factors

**Figure 3:**
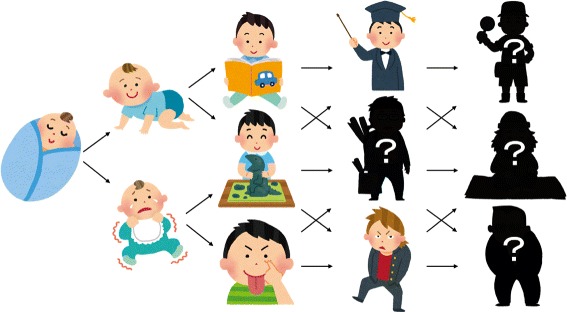
**Example of the growing patterns.** Example character growing patterns which the learners can find when they continue trying some questions.

The first factor is a growth character system. The character shown in Figure 2 (a) and Figure 2 (b) grows depending on the learner’s results, the learning interval, and the duration of learning. Figure 2 (a) in the upper right portion of the screen is growing character, and the first character is a baby. When the learner plays in time trial mode or weak point learning mode, clear points will be gained as illustrated in Figure 2 (c). The clear points are accumulated in the progress bar under the baby image in Figure 2 (a). Finally, when the progress bar is full, the character grows as illustrated in Figure 2 (b). The center character string is a comment about the character image. For example, if the learner does not continue learning, the character goes off the rails, and if the learner continues learning, the character becomes an elite as illustrated in Figure 3.

This is one of the interactive activities in our system for identifying the level of learners’ efforts. The objective of this system is to stimulate learners’ motivation by setting the other objectives such as games.

The second factor is the time trial question and ranking system. In the time trial system, learners can attempt to answer as many questions as possible correctly in a minute. After their attempt, our system shows the clear points depending on the accuracy rate, the number of answered questions, and the difficulty of answered words as illustrated in Figure 2 (c). The information in the middle of Figure 2 (c) indicates a result; the top line indicates the number of answered questions, the second line indicates the percentage of correct answers, the third line indicates difficulty of questions, and the bottom line indicates points gained depending on these results. The learners can gain satisfaction by overcoming their previous score; further, they can feel the improvement of their skills and efforts. Furthermore, this clear points are also reflected in the ranking, which enhances learners’ motivation through their competitive spirit. This application implements the function for weak point learning at the learners’ pace to steadily enhance their skills.

The last factor is SNS connectivity. In this application, learners can submit their clear points to SNS services easily. The learners can satisfy their desire for recognition from others, and this creates a rivalry with others.

### Visualization of learners’ skills

Implementing of gamification is expected to maintain learners’ short-term motivation; however, for keeping their long-term motivation, it is desirable that the learner discovers a pleasure or a purpose of learning in itself. Many learners have difficulty with memorization learning such as English vocabulary learning. In our application, we visualize each learner’s efforts to make the learner’s growth and the increase of knowledge much more recognizable.

#### Visualization of learners’ efforts

This application shows a learner’s efforts such as the number of answered questions, the number of correct answers, and the difficulty of learned questions. The graph upper left in Figure 2 (a) shows the learner’s efforts value that is the sum of the points the learner gained each day in one week.

The learner can confirm the degree of his/her efforts using numeric values and graphs which prevents the decrease of motivation.

#### Visualization of learners’ skill and the degree of memorization

In desk learning, to date, it has been a problem that learners have difficulty in understanding their improvements

: how many words the learner can answer correctly, in this case.

However, utilizing e-learning, which can record and analyze all answers of each learner, our application can visualize the degree of the learners’ mastered knowledge. Our application shows the rate of mastered words and the average rate of correct answers as illustrated in the blackboard at Figure 2 (a). In Figure 1 (c), to visualize the learner’s mastered words and unmastered words, our application shows the degree of memorization for each word by progress bar. The progress bar indicates the percentage of correct answers. If the learner could answer correctly at the last challenge, it indicates full. For example, the progress bar of “abstract” is full because the learner answered it correctly at the last challenge of “abstract”, but the progress bar of “appreciate” is not full because the learner answered it incorrectly at the last challenge of “appreciate”. Our application cannot assess whether the learner has completely memorized each word; therefore, we deemed that the learner memorized the word when the most recent challenge was answered correctly. The progress bar in the upper right portion of Figure 1 (c) is the rate of mastered words, calculated by dividing the number of mastered words by the total number of words. Because the learners will be able to understand their weak points easily, learners can learn efficiently by themselves.

## Question selection by estimating learner’s skills

**Figure 4:**
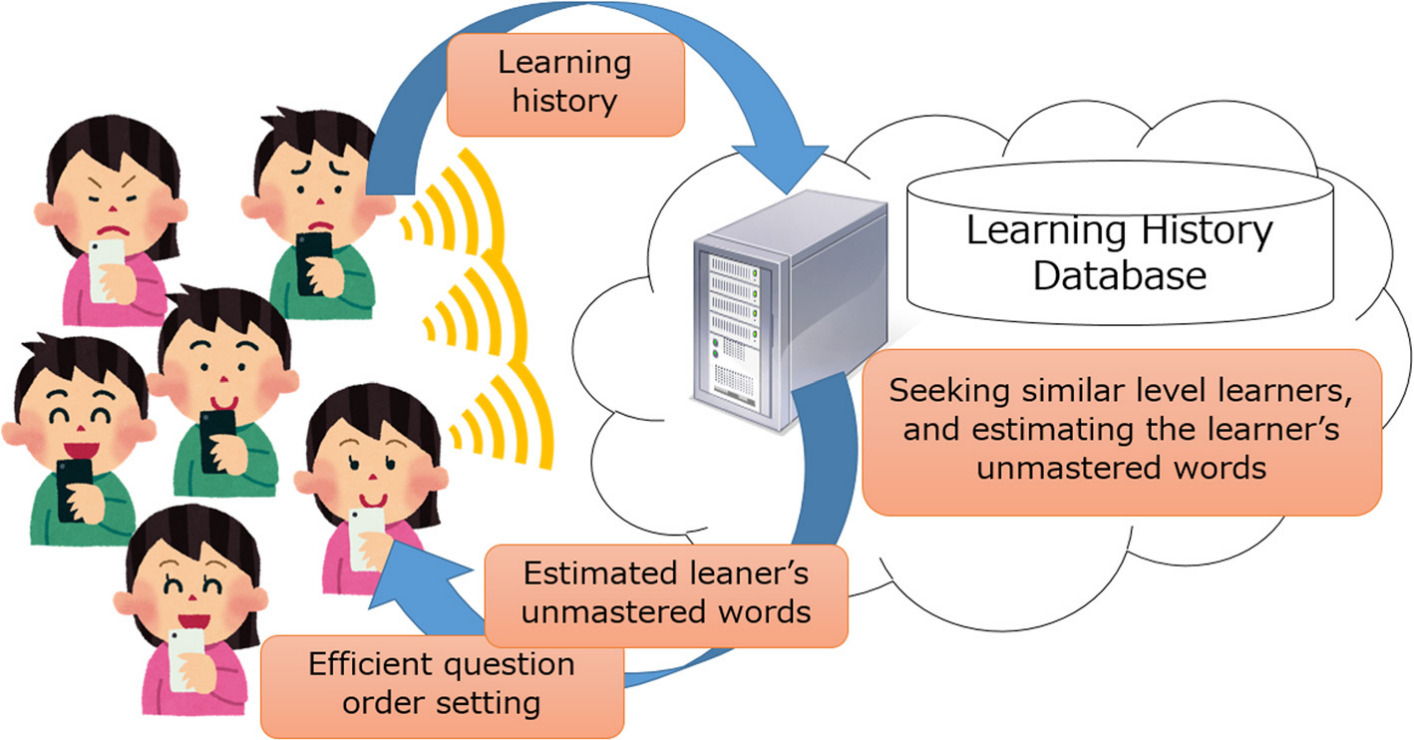
**How to use the cloud in proposed application.** The image of the relationship between proposed application and a cloud.

An advantage of e-learning is that it is easy to collect and analyze learners’ learning histories. Our application collects all learners’ learning histories on a cloud as illustrated in Figure 4 in order to feed back the information for keeping learners’ motivation. In this paper, the information for keeping learners’ motivation is the provision of words of an appropriate difficulty for each learner based on the learner’s estimated skill using learning histories. We describe how difficulty selection affects the learner’s motivation (Bachen and Raphael 2011; Jegers 2007) in the proposal system design section of our paper. Designers need to set the suitable difficulty for an individual player to maintain his/her motivation. Therefore, we considered that learners could continue learning efficiently with high motivation when our application could set questions with a suitable percentage of mastered words and unmastered words. Unfortunately, our application cannot know which words already mastered unless the learner has challenged all words. More specifically, in memorizing 1000 words, the learner has to answer 1000 words even if the learner has already mastered 500 of them. This is a necessary step for memorizing, but it is a huge burden and pains. In our study, we propose a new method that estimates whether the learner has already mastered a word; accordingly, setting a suitable percentage of mastered words and unmastered words improves the learner’s motivation.

### Classifying English words based on the degree of similarity between each learner

In this study, based on the assumption of the similarity between each learner’s mastered or unmastered words, we propose a new method that estimates whether the learner knows unlearned words by the degree of similarity between each learner. Applying the matrix in collaborative filtering technique Herlocker et al. (2000), we considered whether the learners *u*∈{*u*_1_,*u*_2_,*u*_3_,...,*u*_*n*_} knew each English word *w*∈{*w*_1_,*w*_2_,*w*_3_,...,*w*_*m*_} as true (T: the learner knew it) or false (F: the learner did not know it) as shown in Table 1. For example, when estimating whether the learner *u*_*n*_ knew the word *w*_*m*_, we can estimate that *u*_*n*_ knew *w*_*m*_ because *u*_*n*_ is similar to *u*_4_ and *u*_6_.

**Table 1:** **An example of the learning history database on cloud (T:True F:False)**

	***w***_1_	***w***_2_	***w***_3_	***w***_4_	***w***_5_	***w***_6_	**⋯**	***w***_***m***_
*u*_1_	F	T		F		T		F
*u*_2_	T	F		T	T	T		
*u*_3_	T		T	T	T			T
*u*_4_		T		T		T		T
*u*_5_	T		T	T	T	T		T
*u*_6_			F	T	F	T		T
⋯								
*u*_*n*_	T	T	F		F	T		

Our method calculates *Value*(*u*,*w*) based on a weighting average of similarity values by 10 learners’ nearest neighbors. *Value*(*u*,*w*) means the estimated value which indicates whether the learner *u* can answer the word *w* correctly. Rounding *Value*(*u*,*w*) to the nearest whole number, when the rounded value is 1, it means that the learner *u* has already mastered the word *w*. *Similarity*(*u*,*v*) means the degree of similarity between the learner *u* and *v*. These are calculated as follows,

(1) $$ \begin{array}{l} Value\left(u,w\right)=\frac{\sum_{v\in {V}^{\prime }}\left( Similarity\left(u,v\right)\times {r}_{vw}\right)}{\sum_{v\in {V}^{\prime }} Similarity\left(u,v\right)}\end{array} $$

(2) $$ \begin{array}{l} Similarity\left(u,v\right)=\frac{1}{1+\frac{Distance\left(u,v\right)}{\sqrt{\left|{W}^{\prime}\right|}}}\end{array} $$

where *u* is a learner about whom we estimate whether a word *w* has already been mastered, *V*^′^ is a group of 10 learners’ who have the highest degree of similarity of all learners, *r*_*vw*_ is the latest answer for the word *w* by the learner *v* (True is 1 and False is 0), and |*W*^′^| is the number of words which the learner *u* has already learned. *Distance*(*u*,*v*) means the distance between the learner *u* and *v*, and it is calculated by Euclidean Distance according to the following formula.

(3) $$ \begin{array}{l} Distance\left(u,v\right)=\sqrt{\sum_{w\in {W}^{\prime }}{\left({r}_{uw}-{r}_{vw}\right)}^2}\end{array} $$

### The ratio of unmastered questions to maintain the learners’ motivation

All words in our application can be classified as one of the following four kinds: mastered word, unmastered word, estimated mastered word, and estimated unmastered word. Using answer history data, our application can assess whether a learner has already mastered a word when the learner has challenged the word in our application. When the learner could answer the word correctly on the most recent attempt, the word is classified as a mastered word. When the learner could not answer the word correctly at the last time, the word is classified as an unmastered word. Using the above-mentioned method estimates whether the learner can answer the word correctly. When our method estimates true, the word is classified as an estimated mastered word. When our method estimates false, the word is classified as an estimated unmastered word. It is generally said that a rate of over 80 % known words in long sentences is good for sustainable motivation when reading long English books. Our application sets questions with the same ratio of the above-mentioned four kinds of words because our goal is to support English vocabulary learning with sustainable motivations; therefore, the ratio of mastered words is 25%, the ratio of unmastered words is 25%, the ratio of estimated mastered words is 25%, and the ratio of estimated unmastered words is 25%. Because unmastered words have already appeared one or more times despite incorrect answers at the most recent attempt, assuming that the learner knows unmastered words, the sum of the ratio of the known words is 75%. The learner can review and learn new words in this ratio with sustainable motivation.

### Experience to evaluate accuracy

**Figure 5:**
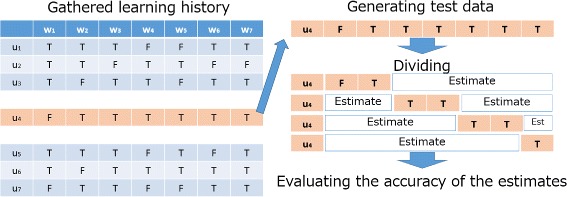
**How to evaluate the accuracy of the proposed estimate method.** How to evaluate the accuracy: (1) Select a row, (2) Divide the selected data, (3) Estimate each blank space, (4) Evaluate the accuracy of estimated result.

To evaluate the estimation accuracy, we collected 105 questions’ answers by 53 participants using a developed web application ^c^ which has 105 questions included in our application. We evaluated the estimation accuracy using a cross-validation method for each learner as illustrated in Figure 5: (1) extract an answered record as test data from gathered learning data; (2) divide the selected data to generate test data; (3) estimate each blank space; (4) check whether the estimated data equals a correct answer; (5) calculate the estimation accuracy. At this time, the number of partitions is tentatively 10; therefore, for each estimation, using about 10 answered words, about 90 other words will be estimated.

Table 2 indicates the result of our evaluation. Because there are no existing methods having a similar purpose, we compared our proposal accuracy with the case that all estimated answers were true, and the case that all estimated answers were false. In this result, true means that the learner’s answer was correct, and false means that the learner’s answer was incorrect. In Table 2, T is true, and F is false. Table 3 is a cross table between actually answered data and estimated results.

**Table 2:** **A comparison between the recall, precision and F-measure**

	**Accuracy**	**Recall(T)**	**Recall(F)**	**Precision(T)**	**Precision(F)**	**F-measure(T)**	**F-measure(F)**
Proposal	82.7%	95.9%	27.7%	84.6%	61.8%	89.9%	38.3%
All true	80.6%	100.0%	0.0%	80.6%	0.0%	89.3%	0.0%
All false	19.4%	0.0%	100.0%	0.0%	19.4%	0.0%	32.5%

**Table 3:** **The evaluation result**

		**Right data (Acutually answered data)**		
		**True (Correct)**	**False (Incorrect)**	
Estimated Result	True (Correct)	39436	7158	46594
	False (Incorrect)	1694	2742	4436
		41130	9900	51030

Focusing on the accuracy of each, this result indicates that our proposed method could estimate, from 10 extracted words, whether or not about 90 words have already been mastered with 82.7% accuracy. However, because of the bias, if we estimate all results as true, the accuracy was 80.6%. Although the accuracy of our method is a little higher than the acuracy of all true, in terms of F-measure, our proposal both F-measure (T) and (F) was better than them.

## Our application evaluation

Although we should compare the tendency of the learner’s learning continuation between our proposed system and other e-learning systems, it is difficult to compare the tendency under the same conditions. We performed a questionnaire to consider our application’s evaluation and its effects through actual uses by twenty-seven participants. The participants are composed of undergraduates and graduate students whose age is between 19 and 24. Participants answered the questionnaire for each function on a scale of one to five: five means “Strongly agree”, four means “Agree”, three means “Neither agree nor disagree”, two means “Disagree”, and one means “Strongly disagree.” We show the contents of the questionnaire and its results in Table 4 where the results are composed of three scales; “Agree” includes answers five and four, “Neither agree nor disagree” is answer three, and “Disagree” includes answers two and one.

**Table 4:** **Contents of the questionnaire and their results**

**Function**	**Content**	**Agree**	**Neither agree nor disagree**	**Disagree**
Weak point learning	1. I may learn on this app with sustainable motivation.	16 (59.3%)	10 (37.0%)	1 (3.7%)
	2. I enjoyed playing this app.	14 (51.9%)	11 (40.7%)	2 (7.4%)
	3. I felt that I could memorize English words.	8 (29.6%)	13 (48.1%)	6 (22.2%)
	4. I want to play this app once again.	11 (40.7%)	12 (44.4%)	4 (14.8%)
Time Trial Challenge	5. I may learn on this app with sustainable motivation.	17 (63.0%)	8 (29.6%)	2 (7.4%)
	6. I enjoyed playing this app.	18 (66.7%)	5 (18.5%)	4 (14.8%)
	7. I felt that I could memorize English words.	6 (22.2%)	13 (48.1%)	8 (29.6%)
	8. I want to play this app once again.	12 (44.4%)	12 (44.4%)	3 (11.1%)
Character growth	9. I may learn on this app with sustainable motivation.	12 (46.2%)	7 (26.9%)	7 (26.9%)
	10. I want to grow the character more.	14 (53.8%)	4 (15.4%)	8 (30.8%)
BPQ50792-xml0x.pngCleRaranpkioinngts\c:IMG	11. I may learn on this app with sustainable motivation.	19 (73.1%)	5 (19.2%)	2 (7.7%)
	12. I want to play this app more times to raise my score.	16 (61.5%)	5 (19.2%)	5 (19.2%)
SNS	13. I may learn on this app with sustainable motivation.	6 (23.1%)	10 (38.5%)	10 (38.5%)
	14. I may submit my score when I get a good score.	5 (19.2%)	5 (19.2%)	16 (61.5%)

According to these results, many participants answered “Agree” for much of the content; however, we focus on some distinguishing points. First, between content no.2 and no.6, time trial challenge receive more “Agree” responses than weak point learning; however, it also receive more “Disagree” responses. We considered that the learners felt that time trial challenge was more characteristic than weak point learning because the number of answers of “Neither agree nor disagree” in the time trial challenge was significantly fewer than that in the weak point learning. Therefore, time trial challenge has a good influence on learners’ sustainable motivation for some learners. Second, about content no.11, the ratio of “Agree” was 73.1% which was the highest ratio of all; therefore, most learners are especially interested in the clear points and ranking function. Finally, about content no.13 and no.14, both these contents have a lower ratio of “Agree”; further, the ratio of “Disagree” in no.14 was 61.5% which was the highest ratio of all; therefore, most learners were not interested in submitting their score to SNS services, and they thought that the SNS function did not affect their motivation in this case.

Because our application got many “Agree” answers, we considered that the gamification techniques had a good influence in some functions. In this paper, we found out that learners were especially interested in the clear points and ranking function, and were not interested in SNS submitting. We do not believe that the SNS function is not good for sustainable motivation, but we believe that a suitable utilization of SNS supports learners’ motivation; therefore, we should consider how to utilize SNS services to maintain learners’ motivation in future efforts.

## Conclusion

In this study, focusing on motivated learners who cannot continue learning, we developed an English vocabulary learning support system for sustainable motivation. This application is equipped with the necessary functions for English vocabulary learning. In addition, we utilized information technology, such as gamification techniques, cooperation with SNS, and an unlearned words estimation based on the degree of similarity between each learner. Therefore, we believe that we have succeeded in making an application that supports the growth of self-sufficient learners in the following steps: (1) learner who cannot continue learning; (2) learner who enjoys our application in terms of gamification; (3) learner who is interested in self-growth; (4) self-sufficient learner.

This application is currently available on Android platforms 2.2 and above; further it is published in Google Play. As future efforts, we will evaluate the degree of continuation using our application. Moreover, to improve our proposed estimation accuracy, after gathering more usage data, we will consider parameters and estimation algorithms.

## Endnotes

^a^ Foursquare https://foursquare.com/

^b^ Ke-Tan for TOEIC basic vocablary: https://play.google.com/store/apps/details?id=com.has.seelearning

^c^ TOEIC vocabulary level check Vol.1 http://t-hase.rhcloud.com/
